# Fetal genome predicted birth weight and polycystic ovary syndrome in later life: a Mendelian randomization study

**DOI:** 10.3389/fendo.2023.1140499

**Published:** 2023-06-07

**Authors:** Dong Liu, Yuexin Gan, Yue Zhang, Linlin Cui, Tao Tao, Jun Zhang, Jian Zhao

**Affiliations:** ^1^Ministry of Education and Shanghai Key Laboratory of Children’s Environmental Health, Xinhua Hospital, Shanghai Jiao Tong University School of Medicine, Shanghai, China; ^2^Department of Bioinformatics and Biostatistics, School of Life Sciences and Biotechnology, Shanghai Jiao Tong University, Shanghai, China; ^3^Center for Reproductive Medicine, The Second Hospital, Cheeloo College of Medicine, National Research Center for Assisted Reproductive Technology and Reproductive Genetics, Shandong University, Jinan, Shandong, China; ^4^Department of Endocrinology and Metabolism, Renji Hospital, School of Medicine, Shanghai JiaoTong University, Shanghai, China; ^5^Department of Maternal and Child Health, School of Public Health, Shanghai Jiao Tong University, Shanghai, China; ^6^Medical Research Council (MRC) Integrative Epidemiology Unit, University of Bristol, Bristol, United Kingdom

**Keywords:** Mendelian randomization, birth weight, polycystic ovary syndrome, fetal genome, genetic pleiotropy

## Abstract

Associations between lower birth weight and higher polycystic ovary syndrome (PCOS) risk have been reported in previous observational studies, however, the causal relationship is still unknown. Based on decomposed fetal and maternal genetic effects on birth weight (n  =  406,063), we conducted a two-sample Mendelian randomization (MR) analysis to assess potential causal relationships between fetal genome predicted birth weight and PCOS risk using a large-scale genome-wide association study (GWAS) including 4,138 PCOS cases and 20,129 controls. To further eliminate the maternally transmitted or non-transmitted effects on fetal growth, we performed a secondary MR analysis by utilizing genetic instruments after excluding maternally transmitted or non-transmitted variants, which were identified in another birth weight GWAS (n = 63,365 parent-offspring trios from Icelandic birth register). Linkage disequilibrium score regression (LDSR) analysis was conducted to estimate the genetic correlation. We found little evidence to support a causal effect of fetal genome determined birth weight on the risk of developing PCOS (primary MR analysis, OR: 0.86, 95% CI: 0.52 to 1.43; secondary MR analysis, OR: 0.86, 95% CI: 0.54 to 1.39). In addition, a marginally significant genetic correlation (r_g_ = -0.14, se = 0.07) between birth weight and PCOS was revealed *via* LDSR analysis. Our findings indicated that observed associations between birth weight and future PCOS risk are more likely to be attributable to genetic pleiotropy driven by the fetal genome rather than a causal mechanism.

## Introduction

Polycystic ovary syndrome (PCOS), affecting 6% – 9% of women of reproductive age, is the most common endocrine condition (1). Based on previous studies, insulin resistance, obesity, and androgen excess may contribute together and play crucial roles in PCOS development (2, 3). In addition, an increasing body of evidence suggests a strong genetic component in its aetiology (4, 5). However, the aetiology of PCOS remains largely unknown, and no efficient therapeutic treatments or prevention measures for PCOS are available. According to the Developmental Origins of Health and Disease (DOHaD) hypothesis, early life abnormal growth and development were associated with the risk of developing various chronic diseases in later life (6, 7). Birth weight, a common indicator reflecting intrauterine fetal growth, has been widely studied on its long-term impact on adulthood health outcomes (8–11). Interestingly, observational associations between birth weight and PCOS risk in later life have been reported in a recent meta-analysis and multiple cohort studies (12–17). However, these associations were not well replicated in other independent large-scale cohort studies (18–20). Given that observational studies are commonly prone to residual confounding or reverse causation (21), the causal relationship between birth weight and the risk of developing PCOS remains unknown.

Mendelian randomization (MR), which is a causal inference technique using genetic variants randomly allocated during conception as instrumental variables, is less prone to residual confounding or reverse causation bias (22). In a previous study, little evidence was found to support a causal effect of birth weight on PCOS risk by using MR (P = 0.22) (23). However, this study used offspring genetic variants associated with birth weight as instrumental variables without adjusting for maternal genotypes, which were correlated with fetal genotypes (r ≈ 0.5) (24, 25) (**Supplemental Figure 1**). Thus, their effect estimates of birth weight on PCOS risk might be biased by the maternal genetic effects. In addition, recent studies suggested that composite or complex traits can be explained by multiple components or distinct biological pathways (26–28). Like other complex traits, variation in birth weight can also be explained by different components, such as fetal genetically regulated components and maternal adverse intrauterine environment components (29–31). Dissecting these components of birth weight is essential to understand the underlying biological mechanism. Recently, several studies investigated possible mechanisms between birth weight and cardiometabolic risk by using different components of birth weight. Based on structural equation model (SEM) and weighted linear model (WLM) methods, Warrington et al. and Moen et al. recently separated genetic effects on birth weight into maternal and fetal components to investigate the causal mechanisms between birth weight and future cardiometabolic risk (29, 32). Their findings suggested that associations between birth weight and adulthood cardiometabolic outcomes were attributable to fetal genetic effects rather than intrauterine programming (29, 32). Moreover, from a genomic perspective, Juliusdottir et al. discriminated the effects of transmitted and non-transmitted alleles on birth weight by using a long-range phasing (LRP) method based on the Icelandic fetal growth samples to investigate inheritance patterns affecting birth weight (30). This study indicated that associations between birth weight and most cardiometabolic risk factors were driven by the fetal genome (30), whereas it is still unclear whether birth weight affects PCOS in the same manner.

Recently, two large-scale genome-wide association study (GWAS) meta-analyses on PCOS released their summary statistics (4, 33), which provided opportunities for assessing potential causal relationships between birth weight and PCOS risk. Thus, in this study, we aimed to investigate whether there is a causal effect of fetal genome determined birth weight on PCOS risk using two-sample MR analysis. Considering other potential mechanisms that might underpin the association between birth weight and PCOS, such as genetic pleiotropy, we also assessed the genetic correlation between birth weight and PCOS risk by conducting linkage disequilibrium score regression (LDSR) analysis which is mainly used to identify shared genetic variation between two traits across the whole genome (34, 35).

## Materials and methods

### Data sources and study populations

**Figure 1:**
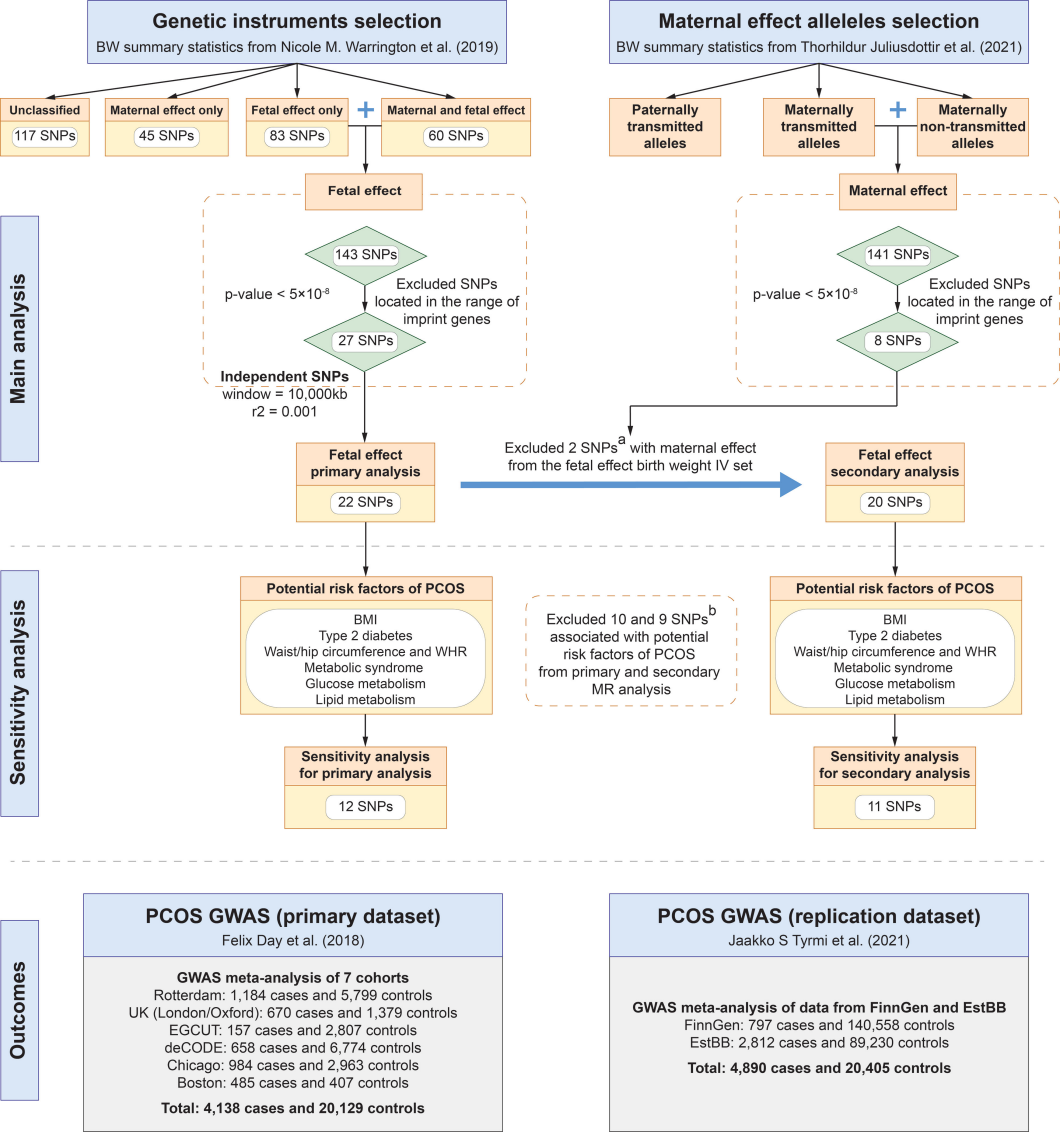
Study design of MR analyses. **(A)** rs560887 and rs10872678 were identified as maternally transmitted and non-transmitted alleles respectively in the birth weight GWAS by Juliusdottir et al. (30). **(B)** SNPs were genome-wide significantly associated with potential confounders of PCOS, including BMI, type 2 diabetes, waist/hip circumference, waist-to-hip ratio, metabolic syndrome, glucose metabolism, and lipid metabolism. BMI, body mass index; BW, birth weight; EstBB, Estonian Biobank; GWAS, genome-wide association study; PCOS, polycystic ovary syndrome; SNP, single nucleotide polymorphism; WHR, waist-to-hip ratio.

A schematic overview of the study design is presented in **Figure 1** and detailed data sources information can be found in **Supplemental Table 1**. We used two sets of birth weight summary statistics obtained from GWASs conducted by the Early Growth Genetics (EGG) consortium (http://egg-consortium.org) and the Icelandic birth register to construct two sets of instrumental variables (IVs) for the primary and secondary MR analysis, respectively. GWAS of birth weight conducted by the EGG Consortium included 406,063 individuals of European ancestry (29), where maternal and fetal genetic effects on birth weight were separated by using SEM. In the primary MR analysis, we used the summary statistics of fetal genetic effects on the offspring’s birth weight after adjusting for correlated maternal genotypes. Of note, the original birth weight GWAS categorized 305 genome-wide significant (P < 5×10^-8^) single nucleotide polymorphisms (SNPs) identified into 5 groups based on the effects of maternal and/or fetal genotypes on offspring birth weight: 1) fetal effect only, 2) maternal effect only, 3) fetal and maternal effects with the same direction, 4) fetal and maternal effects with the opposite directions, and 5) unclassified (29). Among these variants, 28 SNPs were identified as having fetal genetic effects on birth weight (SEM classification: “fetal only” or “fetal and maternal”).

The outcome data were obtained from a large-scale GWAS meta-analysis of PCOS, including 4,138 cases and 20,129 controls of European ancestry from six cohorts (Rotterdam, Oxford, EGCUT, deCODE, Chicago, and Boston) (4). To further validate the results of the MR analysis, we used summary statistics of PCOS GWAS meta-analysis in the FinnGen and Estonian Biobank (EstBB) as replication data, which included 3,609 cases and 229,788 controls (33).

### Genetic instruments selection

The genetic instruments selection procedure was conducted in the following steps. First, in the primary MR analysis, statistically significant (P < 5×10^-8^) genetic variants were selected from summary statistics of birth weight GWAS conducted by the EGG Consortium (29). To ensure that genetic variants are independent, a stringent linkage disequilibrium (LD) threshold (r^2^ < 0.001 and window size = 10,000 kb) was used for LD clumping, with the European subsample of 1,000 Genome Project data as reference panel (36). Moreover, we excluded genetic instruments located in the range of imprinted genes to minimize the heterogeneous effect of variants on phenotypes in the population. Considering potential violations of the MR core assumptions, that is, maternal genetic effects confounded fetal genetic variants which were used as IVs and the outcome (i.e., PCOS), we identified and excluded SNPs that exerted maternal genetic effects on birth weight from the set of IVs. To further eliminate maternal genetic effects on birth weight from IVs used in the primary analysis, we identified and excluded maternally transmitted and non-transmitted alleles based on a GWAS meta-analysis on birth weight by Juliusdottir et al. from 63,365 parent-offspring trios (30), to construct IVs for the secondary MR analysis. The allele-specific effects of maternally transmitted or non-transmitted on birth weight were used to represent the maternal and fetal genetic effects, respectively (37). Finally, five maternally transmitted and three maternally non-transmitted SNPs that reached a genome-wide significant level on birth weight were identified from the GWAS by Juliusdottir et al. (30)

Furthermore, we extracted SNP-POCS associations for each genetic instrument from two independent PCOS GWASs conducted by Day et al. and Tyrmi et al., respectively (4, 33). If a certain instrument was not available in the summary data, a proxy SNP in high LD in the European population was identified using LDlink (https://ldlink.nci.nih.gov/?tab=ldproxy). After that, data harmonization was performed to combine SNP-birth weight and SNP-PCOS associations using the “harmonise_data” function in the TwoSample MR package (36), in which ambiguous or palindromic SNPs were excluded.

As a result, we retained a total of 22 SNPs as genetic instruments in the primary MR analysis from birth weight GWAS conducted by Warrington et al. (29) and 20 SNPs after excluding two maternally transmitted or non-transmitted SNPs (i.e., rs560887 and rs10872678 which were identified in the GWAS by Juliusdottir et al. (30)) in the secondary MR analysis (**Table 1**). To minimize the risk of violating the IV assumptions, we identified SNPs associated with risk factors for PCOS, including body mass index (BMI), type 2 diabetes, waist/hip circumference, waist-to-hip ratio, metabolic syndrome, glucose metabolism, and lipid metabolism, by searching the GWAS Catalog database (https://www.ebi.ac.uk/gwas/) and the PhenoScanner database (version 2; http://phenoscanner.medschl.cam.ac.uk/). After excluding associated SNPs, 12 and 11 SNPs were retained as genetic instruments in each set of IVs, respectively (**Figure 1**, **Supplemental Tables 2, 3**).

**Table 1:** Characteristics of instrumental variables for birth weight used in the primary MR analysis.

SNP	CHR	Position	Gene	EA	OA	EAF	Beta	SE	P	F^*^
rs80278614	1	119412317	*TBX15*	A	G	0.05	0.05	0.009	4.03×10^-8^	30.1
rs2551347	2	23912401	*KLHL29*	T	C	0.75	0.03	0.005	2.20×10^-9^	35.8
rs17034876	2	46484310	*EPAS1*	T	C	0.70	0.04	0.005	5.47×10^-17^	70.2
rs560887 ^a,b^	2	169763148	*G6PC2*	C	T	0.70	-0.02	0.004	2.78×10^-8^	30.9
rs11708067 ^b^	3	123065778	*ADCY5*	G	A	0.25	0.06	0.005	6.26×10^-32^	138.3
rs1482852 ^b^	3	156798294	*LOC339894*	A	G	0.60	0.05	0.004	7.56×10^-39^	170.0
rs4144829 ^b^	4	17903654	*LCORL*	C	T	0.26	0.03	0.005	1.12×10^-11^	46.1
rs35261542 ^b^	6	20675792	*CDKAL1*	C	A	0.74	0.05	0.005	3.23×10^-26^	112.2
rs10872678 ^a^	6	152039964	*ESR1*	T	C	0.72	0.03	0.005	8.23×10^-10^	37.7
rs138715366	7	44246271	*YKT6/GCK*	C	T	0.99	0.24	0.022	1.43×10^-25^	109.3
rs112139215	7	73034559	*MLXIPL*	A	C	0.07	0.06	0.008	1.20×10^-11^	46.0
rs13266210	8	41533514	*ANK1*	A	G	0.78	0.03	0.005	3.05×10^-9^	35.2
rs28457693	9	98217348	*PTCH1*	G	A	0.11	0.04	0.007	1.70×10^-9^	36.3
rs1112718 ^b^	10	94479107	*HHEX/IDE*	G	A	0.41	0.04	0.004	1.51×10^-17^	72.7
rs7076938 ^b^	10	115789375	*ADRB1*	T	C	0.73	0.03	0.005	2.91×10^-10^	39.7
rs4444073	11	10331664	*ADM*	A	C	0.51	0.02	0.004	2.20×10^-8^	31.3
rs7968682 ^b^	12	66371880	*HMGA2*	G	T	0.49	0.04	0.004	4.87×10^-20^	84.0
rs75844534	15	38667117	*SPRED1*	A	C	0.12	0.04	0.006	1.54×10^-8^	32.0
rs7402983 ^b^	15	99193276	*IGF1R*	A	C	0.41	0.03	0.004	4.61×10^-10^	38.8
rs222857	17	7164563	*CLDN7*	T	C	0.57	0.03	0.004	5.77×10^-10^	38.4
rs11698914	20	31327144	*COMMD7*	C	G	0.23	0.03	0.005	2.75×10^-9^	35.3
rs1012167 ^b^	20	39159119	*MAFB*	C	T	0.41	0.02	0.004	1.86×10^-8^	31.6

* The selected instruments explain 0.3% of the variation in birth weight in the primary MR analysis. The F statistic of individual SNPs ranged from 30.1 to 170.0 with an average F statistic of 58.2.

a . Maternally transmitted or non-transmitted alleles were excluded from the secondary MR analysis.

b . SNPs were genome-wide significantly associated with potential confounders of PCOS, including BMI, type 2 diabetes, waist/hip circumference, waist-to-hip ratio, metabolic syndrome, glucose metabolism, and lipid metabolism.

BMI, body mass index; CHR: chromosome; EA: effect allele; EAF: effect allele frequency; OA: other allele; P, N, and F indicate p-value, sample size, and F statistic, respectively; SE: standard error; SNP: single-nucleotide polymorphism.

### Primary MR analysis

#### Main analysis

The multiplicative random-effects inverse-variance weighted (IVW) method was used as the main analysis (38, 39). Wald ratio estimate for each SNP was calculated by dividing the per allele effect on PCOS by the per allele change in the standard deviation (SD) of birth weight, followed by meta-analyzing the estimates *via* the multiplicative random-effects IVW method, which eventually yielded the IVW estimates. The IVW estimates can be interpreted as the odds ratio (OR) of PCOS risk for one SD change in birth weight.

### Sensitivity analyses

#### Assessment of the IV assumptions

To test the MR relevance assumption (i.e., whether the selected IVs have strong associations with birth weight), the F statistic was calculated for each genetic instrument in our study (40). Furthermore, to ensure that the exclusion restriction assumption holds, Cochran’s Q statistic in the IVW analysis (38, 39) was used to assess the heterogeneity of the causal estimates between genetic variants (41). The intercept term of MR-Egger regression was used to test for directional pleiotropy. In addition, we conducted the leave-one-out (LOO) (42) and the Mendelian Randomization Pleiotropy RESidual Sum and Outlier (MR-PRESSO) (43) analyses to detect strong influential SNPs or outliers.

### Robust MR methods

Given that the IVW method provides a biased estimate in the presence of unbalanced horizontal pleiotropy (i.e., directional pleiotropy), we carried out sensitivity analyses by using several pleiotropic-robust methods, including MR-Egger (44), weighted median (45), weighted mode (46), and MR-PRESSO (43) methods, to enhance the robustness of causal inference. When the assumption of the Instrument Strength Independent on Direct Effect (InSIDE) holds, the MR-Egger regression will generate consistent estimates even in the presence of directional pleiotropy (47). The assumption of InSIDE allows for the pleiotropy effects of IVs but requires that the SNP-exposure effects are independent of the pleiotropic effects of SNPs on the outcome, which is a weaker assumption than the IVW assumption. However, the MR-Egger estimate is less precise than the IVW estimate, particularly when the SNP-exposure effect estimates of each genetic variant are relatively homogeneous. Furthermore, we conducted the weighted median analysis which provides reliable estimates when up to 50% of the weight comes from valid IVs. We also carried out the weighted mode analysis which assumes that the most common effect estimate is a consistent estimate of the true effect and allows the majority of variants to be invalid (46). Finally, MR-PRESSO analysis was conducted to estimate the causal effect after correcting for horizontal pleiotropy by removing outliers (43).

### Secondary and replication MR analysis

A secondary MR analysis was conducted using the fetal genetic associations extracted from the birth weight GWAS by Warrington et al. (29), after excluding maternally transmitted or non-transmitted alleles that were identified in the GWAS by Juliusdottir et al. (30) In addition, to validate the causal estimates in the primary MR analysis, a replication MR analysis was performed using data from an independent PCOS GWAS meta-analysis in the FinnGen and EstBB (33). To increase the statistical power and precision of causal estimates, a fixed-effect meta-analysis was conducted to pool the IVW estimates from the primary/secondary and replication analyses.

### LDSR analysis

LDSR analysis was conducted to assess the genetic correlation between offspring birth weight and PCOS risk by using the fetal genetic associations with birth weight after adjusting for maternal genotypes. First, we conducted LDSR analysis based on summary statistics from birth weight GWAS conducted by Warrington et al. (29) and PCOS GWAS conducted by Day et al. (4) For replication, LDSR analysis was performed based on the summary statistic from another independent PCOS GWAS conducted by Tyrmi et al. (33) The heritability of a single trait or the genetic correlation between two traits can be estimated using LDSR analysis based on the LD structure of a reference panel. Unlike MR, LDSR analysis assesses the genetic correlation between two traits by using genetic variants from the whole genome rather than the causal effect between two traits.

All statistical analyses were conducted using the R packages “TwoSampleMR”, “MRPRESSO” and “meta” in R software, version 4.0.0 (R Foundation for Statistical Computing, Vienna, Austria). LDSR analysis was performed using the LDSC software, version 4.0.0 (https://github.com/bulik/ldsc) (34, 35).

## Results

### Main analysis

**Figure 2:**
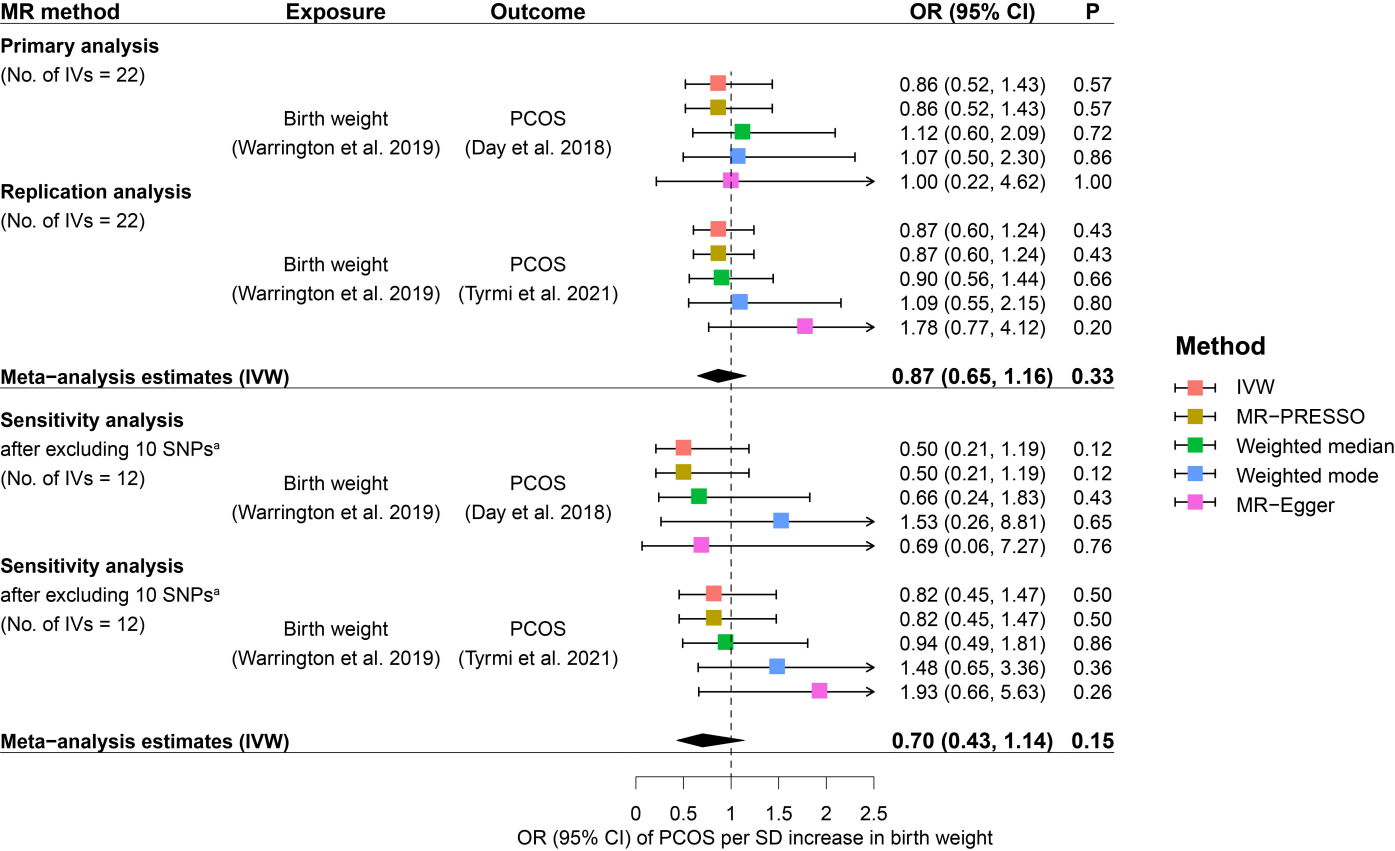
Causal effects of fetal genome determined birth weight on future PCOS risk estimated in the primary MR analysis. Squares represent ORs of PCOS per SD increase in birth weight. Error bars represent 95% confidence intervals. A. 10 SNPs that were genome-wide significantly associated with potential confounders of PCOS, including BMI, type 2 diabetes, waist/hip circumference, waist-to-hip ratio, metabolic syndrome, glucose metabolism, and lipid metabolism, were excluded from the MR analysis. BMI, body mass index; CI, confidence interval; IVs, instrumental variables; IVW, inverse variance weighted; MR, Mendelian randomization; MR-PRESSO, Mendelian Randomization Pleiotropy RESidual Sum and Outlier; OR, odds ratio; P, p-value; PCOS, polycystic ovary syndrome; SD, standard deviation; SNP, single nucleotide polymorphism.

**Figure 3:**
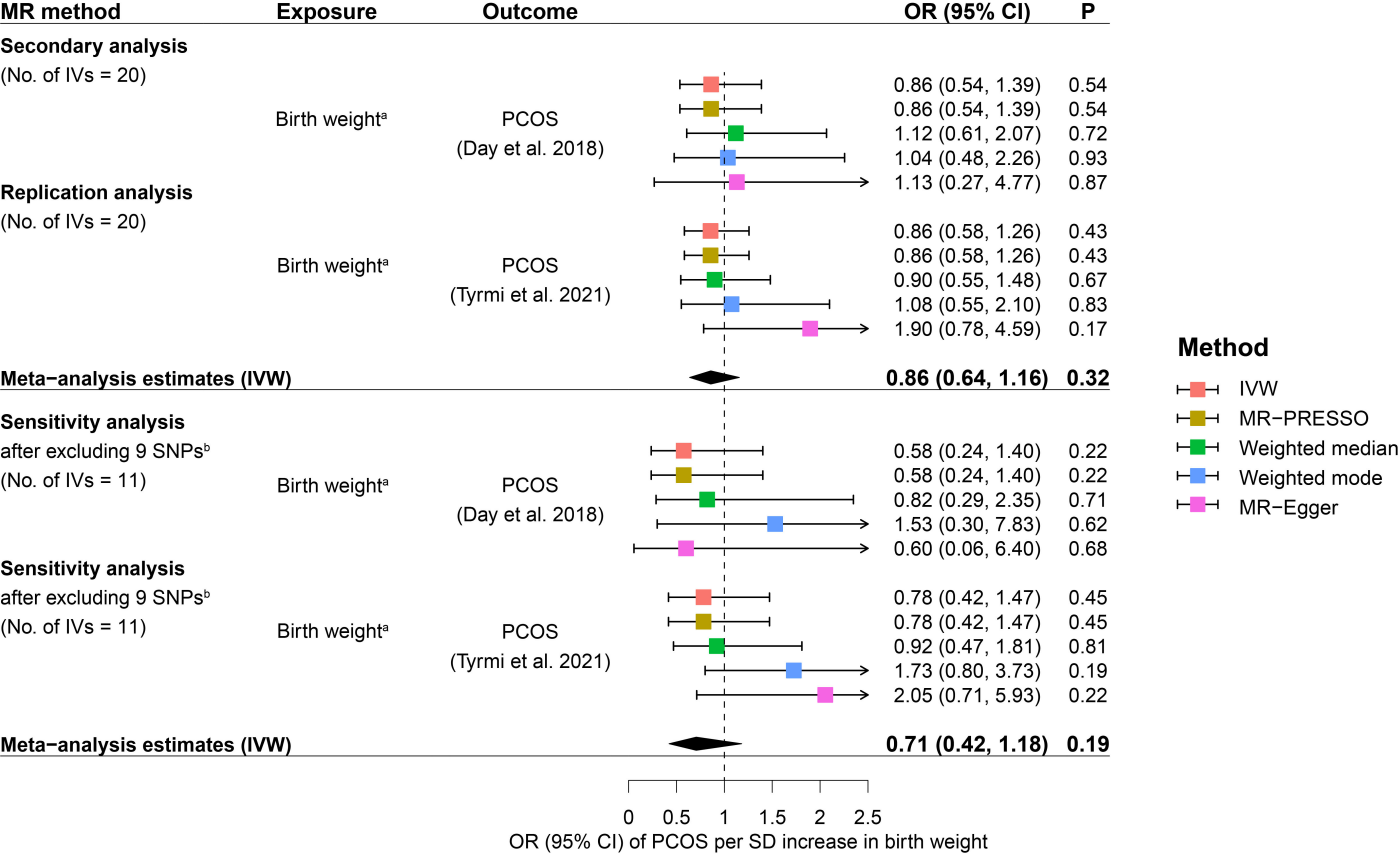
Causal effects of fetal genome determined birth weight on future PCOS risk estimated in the secondary MR analysis. Squares represent ORs of PCOS per SD increase in birth weight. Error bars represent 95% confidence intervals. **(A)** rs560887 and rs10872678were identified as maternally transmitted and non-transmitted alleles respectively in the birth weight GWAS by Juliusdottir et al. (30). **(B)** 9 SNPs that were genome-wide significantly associated with potential confounders of PCOS, including BMI, type 2 diabetes, waist/hip circumference, waist-to-hip ratio, metabolic syndrome, glucose metabolism, and lipid metabolism, were excluded from the MR analysis. BMI, body mass index; CI, confidence interval; IVs, instrumental variables; IVW, inverse variance weighted; MR, Mendelian randomization; MR-PRESSO, Mendelian Randomization Pleiotropy RESidual Sum and Outlier; OR, odds ratio; P, p-value; PCOS, polycystic ovary syndrome; SD, standard deviation; SNP, single nucleotide polymorphism.

The main analysis by IVW suggested little evidence to support a causal relationship between fetal genome determined birth weight and PCOS risk. Causal effect estimates of fetal genome determined birth weight on PCOS risk in the primary MR analysis equated to an OR of PCOS of 0.86 (95% CI: 0.52 to 1.43) for one SD increase in birth weight (**Figure 2**). Replication analysis using another independent data source from PCOS GWAS meta-analysis generated a consistent causal association of fetal genome determined birth weight with offspring PCOS risk (OR: 0.87, 95% CI: 0.60 to 1.24). Further, consistent estimates (regarding both effect directions and magnitudes) were obtained after meta-analyzing the IVW estimates (OR: 0.87, 95% CI: 0.65 to 1.16) in the primary and replication analyses (**Figure 2**). After excluding the maternally transmitted and non-transmitted effects, the MR analysis results suggested a null causal effect of fetal genetically predicted birth weight on PCOS risk in both secondary and replication MR analyses (secondary IVW OR: 0.86, 95% CI: 0.54 to 1.39; replication IVW OR: 0.86, 95% CI: 0.58 to 1.26) (**Figure 3**). A similar pooled IVW estimate was observed (OR: 0.86, 95% CI: 0.64 to 1.16).

### Sensitivity analyses

#### Assessment of the IV assumptions

Genetic instruments for fetal genome determined birth weight, including 22 SNPs, ranged from 30.1 to 170.0 with an average F statistic of 58.2, indicating the absence of weak instruments (**Table 1**). No evidence for heterogeneity between SNP specific causal effect estimates was found for the primary IVs set (*P* for Cochran Q heterogeneity test = 0.10, replication: P = 0.21) and the secondary IVs set (P = 0.26, replication: P = 0.15), respectively. The proximity of the intercept to the origin in the scatter plot (**Supplemental Figure 2**) and no significant difference of the intercept from zero in MR-Egger regression suggested little evidence for directional pleiotropy (primary IVs set: P = 0.85, replication: P = 0.08; secondary IVs set: P = 0.70, replication: P = 0.07) (**Supplemental Table 4**). Meanwhile, the LOO analysis did not detect influential genetic instruments for birth weight in the MR analysis (**Supplemental Figure 3**). The MR-PRESSO global test did not detect any outliers (primary IVs set: P = 0.13, replication: P = 0.23; secondary IVs set: P = 0.30, replication: P = 0.15) (**Supplemental Table 5**). For sensitivity analyses by using IVs after excluding potential confounder-related SNPs, we found little evidence for heterogeneity between the causal effect estimates for each SNP, directional pleiotropy, and any outliers.

### Results from robust MR methods

The results from robust MR methods are presented in **Figures 2** and **3** which were broadly consistent with the IVW analysis results. For the primary IVs set consisting of 22 SNPs, non-significant causal effects of fetal genome determined birth weight on PCOS risk were observed by using MR-PRESSO, weighted median, weighted mode, and MR-Egger methods, respectively (**Figure 2**). Consistent causal effect estimates derived from robust MR analysis were revealed for the secondary IVs set consisting of 20 SNPs (**Figure 3**). The results of sensitivity analysis after excluding potential risk factor related SNPs were consistent with the results of the primary and secondary analyses (**Figures 2**, **3**).

### LDSR analyses

There was a marginally significant genetic correlation (r_g_ = -0.14, se = 0.07, P = 0.05) between birth weight and PCOS on a genome-wide scale. Although the result of replication LDSR analysis showed a non-significant genetic correlation between birth weight and PCOS (r_g_ = -0.16, se = 0.12, P = 0.18), the effect directions and magnitudes were consistent with one another.

## Discussion

In this study, we used MR to test the potential causal relationship between fetal genome predicted birth weight and PCOS risk. From a genomic perspective, it is important to discriminate between the maternally transmitted alleles or intrauterine environment effects and the fetal own genetic effects on birth weight. To confirm our results, we further tested whether there was a causal effect of the fetal genome predicted birth weight on offspring PCOS risk, after excluding maternal transmitted and non-transmitted (i.e., maternal intrauterine environment effects) alleles. Our findings provided little evidence for a causal effect of the fetal genome-determined birth weight on offspring developing PCOS in later life. These findings were consistent with previous observational studies that there was no difference in birth weight between women with PCOS and controls (18–20, 23, 48).

Notably, controversial findings were observed in other studies (12, 49), and the LDSR analysis results of the present study suggested a marginally significant genetic correlation between the two traits. Meanwhile, the potential pleiotropic effects underpinning the link between birth weight and PCOS were reported. A recent study found that two genetic variants (i.e., rs2910164 C > G and rs182052 G > A) in genes *MIR146A* and *ADIPOQ*, both of which were related to PCOS, were associated with birth weight (50). Although a causal effect of birth weight on PCOS risk was not observed in the present MR analysis, genetically pleiotropic effects of variants that contribute to the associations between birth weight and PCOS cannot be ruled out. Our study suggested that the association between birth weight and PCOS is likely to be driven by genetic pleiotropy of variants on the fetal genome.

It is noteworthy that observational studies and animal experiments demonstrated that prenatal exposure to androgens possibly in combination with a genetic predisposition may affect birth weight and subsequent PCOS (51–55). In the present study, the potential confounding of maternal genetic effects was minimized by using fetal genetic variants associated with birth weight as IVs and further excluding maternal transmitted or non-transmitted genetic variants.

### Strengths and limitations

There are several strengths in our study. We benefited from large sample sizes and study design yielding more reliable results. First, to our best knowledge, we used the summary statistics from the largest published birth weight GWAS with adjusting for maternal genetic effects (n = 406,063 European ancestral individuals) to select genetic variants as IVs, and the outcome data were also extracted from the latest or largest GWAS meta-analyses on PCOS (4, 29). Second, as mentioned above, Warrington et al. separated maternal and fetal genetic effects on birth weight by using SEM. We used the fetal genetic effects on their own birth weight as the IV-exposure associations, after adjusting for maternal genetic effects, which could provide insights into the underlying biological or pathogenic mechanisms between fetal growth and PCOS development in later life. Third, we also constructed IVs for birth weight by filtering out maternal transmitted and non-transmitted variants using summary statistics from a study in which the study design is different from the study conducted by Warrington et al. to minimize the confounding bias due to maternal genetic effects. Fourth, we performed a series of sensitivity analyses with multiple sets of IVs and robust MR methods to strengthen the robustness of causal inference. The sensitivity analysis results were consistent with the results of the main analysis.

Several limitations deserve discussion. First, similar to Chen et al. in their description of the methodology, the allele-specific effects on offspring birth weight/fetal growth by maternally non-transmitted, paternally transmitted, and maternally transmitted alleles were used to represent maternal genetic effect, fetal genetic effect, and combination of both, respectively (37). As suggested in the study conducted by Chen et al. (37), we filtered out maternal non-transmitted and transmitted alleles that indicated maternal genetic effects. However, the allele-specific effects on offspring birth weight/fetal growth by maternally transmitted alleles were composed of maternal and fetal effects. In the original study, genetic dissection of maternal and fetal genetic effects was not performed by modeling maternal and fetal effects using linear combinations of these three haplotype effects, that is maternal genetic effect, fetal genetic effect, and a combination of both. Therefore, more large-scale studies are needed to dissect maternal and fetal genetic effects on birth weight using linear combinations of these three haplotype effects in the future. Second, in our study, there exited moderate sample overlap between data on birth weight (in GWAS by EGG Consortium (29)) and PCOS (in GWAS conducted by Day et al. (4)) Up to 2,867 women in the 1958 British Birth Cohort (56) and the Rotterdam Study (57) in the Netherlands National Trial Register (www.trialregister.nl) were included in both GWASs (4, 29). Sample overlap in two-sample MR analysis would bias causal effects estimation (i.e., inflate the false positive rate) (58), whereas in our study null causal effects of birth weight on PCOS risk were revealed in both primary and replication analyses, thus the potential bias due to sample overlap would not alter the conclusion of our findings. Third, PCOS, as a common and complex genetic disease with multiple etiologies, is caused by genes and environmental factors. In the current study, we focus on explaining the genetic correlation between birth weight and PCOS risk. Postnatal environmental effects need to be further tested for with genotypes of father-offspring pairs in the future since paternal genotypes might be associated with offspring PCOS risk after adjusting for offspring genotypes in the presence of postnatal environmental effects. Fourth, previous studies suggested that low birth weight was associated with PCOS development (12), however, other findings supported that women born with extra high birth weight increased the risk of PCOS (15). These inconsistent findings might suggest a non-linear causal effect of birth weight on PCOS risk. The present study was limited by its two-sample MR design and GWAS summary statistics used to assess the potential non-linear effect. It is warranted to be investigated through one-sample MR analysis when individual-level data are available. In addition, for the replication analysis of LDSR, a genetic correlation between birth weight with PCOS did not reach statistical significance. Considering that populations, in which the original GWAS meta-analysis for the replication analysis was conducted, were mainly from the FinnGen and Estonian Biobank (33) that were not fully consistent with populations where the birth weight GWAS was conducted, population stratification might arise. Finally, LD scores estimated from European samples of 1000 Genomes reference data may not represent LD scores well for heterogeneous meta-analyses of GWAS, these may lead to the reduced accuracy of results from LDSR analysis (59). However, both results of genetic correlation based on two different data sets showed an inverse genetic correlation. Therefore, we believe that an inverse genetic correlation between birth weight and PCOS is plausible. To avoid a chance finding, genomic restricted maximum likelihood analysis with individual-level genotype data is needed to further validate our results in the future.

## Conclusions

In conclusion, our findings provided little evidence for a causal effect of fetal genome predicted birth weight on developing PCOS in later life. However, we found evidence for genetic pleiotropy between birth weight and the future PCOS risk, which has the potential to explain the relationship observed in previous observational studies. In this study, although birth weight within the normal range (i.e., 2,500 to 4,000 grams) may not be causally associated with the risk of PCOS in later life, the potential non-linear causal associations between low/high birth weight and PCOS development need to be further investigated. Further, strong evidence for the genetic pleiotropy between fetal-genome predicted birthweight and later life PCOS risk not only suggests a shared genetic basis but provides novel insight into the common intervention and treatment targets for these two phenotypes.

## Data availability statement

The original contributions presented in the study are included in the article/**Supplementary Material**. Further inquiries can be directed to the corresponding authors.

## Ethics statement

Ethical review and approval was not required for the study on human participants in accordance with the local legislation and institutional requirements. Written informed consent to participate in this study was provided by the participants’ legal guardian/next of kin.

## Author contributions

DL, YG, JuZ, and JiZ initiated the study. DL, YG, and JiZ undertook statistical analyses and drafted the manuscript. All authors contributed to the interpretation of analysis results and critical revision of the manuscript. All authors contributed to the article and approved the submitted version.
