# Supplementary material for: Fetal genome predicted birth weight and polycystic ovary syndrome in later life: a Mendelian randomization study
Source: Front Endocrinol (Lausanne). 2023 Jun 7;14:1140499. doi: 10.3389/fendo.2023.1140499 (PMC10282929; doi:10.3389/fendo.2023.1140499)
Supplement: Supplementary file 1 [file DataSheet_1.docx]

**Supplemental figures**

[Supplemental figure 1 Schematic representation for the genetic correlation between birth weight and offspring PCOS risk. 2](#_Toc128737481)

[Supplemental figure 2 Scatter plots visualizing the causal effect estimates of birth weight on PCOS in later life. 3](#_Toc128737482)

[Supplemental figure 3 Leave-one-out plots for the causal estimates of birth weight on PCOS in later life to identify strongly influential SNPs. 4](#_Toc128737483)


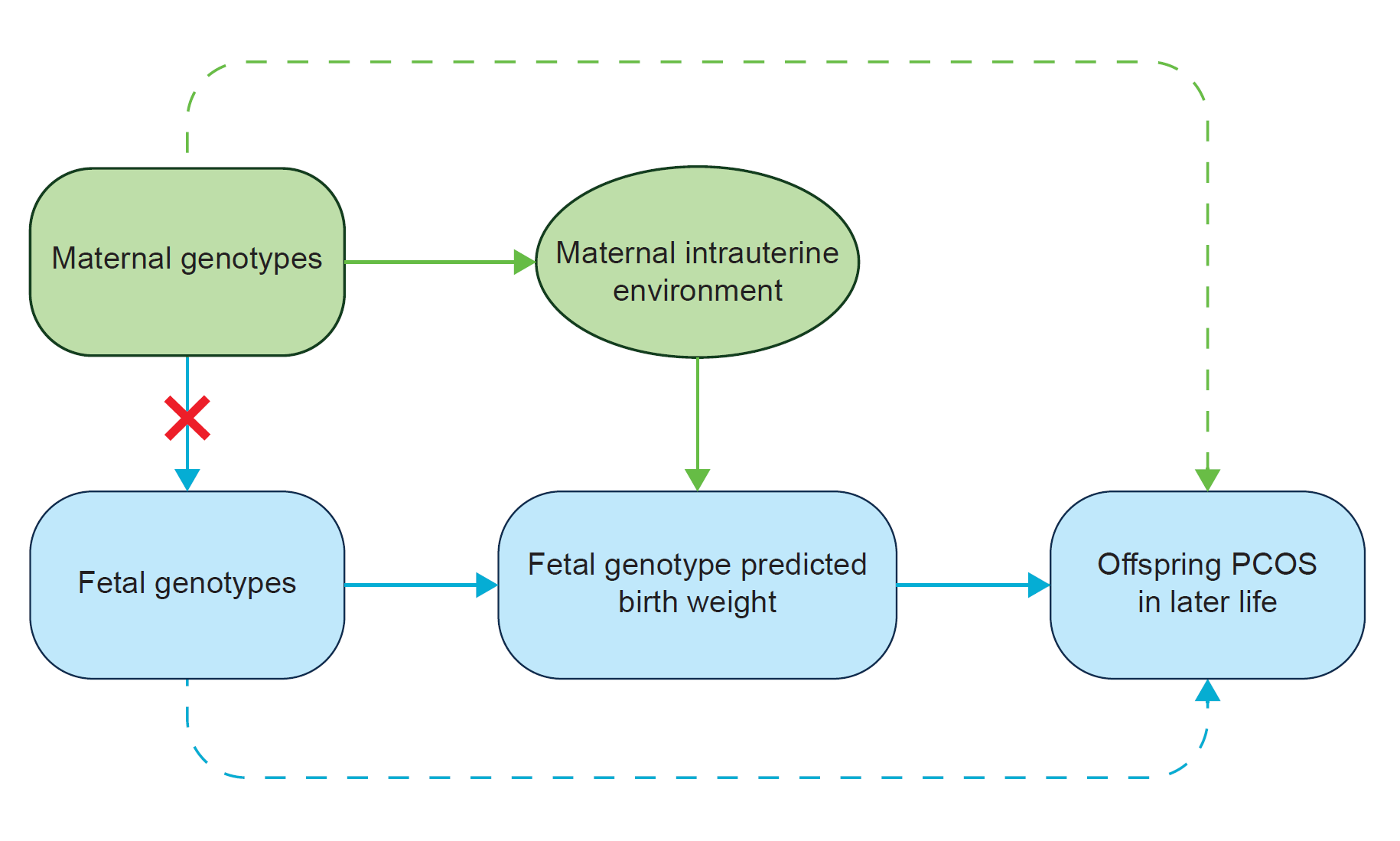


# **Supplemental** figure 1 Schematic representation for the genetic correlation between birth weight and offspring PCOS risk.

Maternal genotypes associated with offspring birth weight yield an adverse intrauterine environment that leads to low offspring birth weight as well as developmental compensations which induce a higher risk of offspring PCOS risk (solid green lines). The inverse genetic correlation between offspring birth weight and offspring PCOS risk is driven by the fetal genotypes which can affect birth weight and further lead to PCOS risk (solid blue lines). There are two other possible mechanisms including 1) maternal genotypes are associated with offspring PCOS risk not via intrauterine environment but via possible postnatal effects of maternal genotypes (dashed green line), and 2) the inverse genetic correlation between both birth weight and offspring PCOS risk was driven by pleiotropic genetic effects of the fetal genome (dashed blue line). The red cross indicates that we are conditioning on maternal genotypes when investigating the causal effect of birth weight on future PCOS risk.

PCOS: polycystic ovary syndrome.


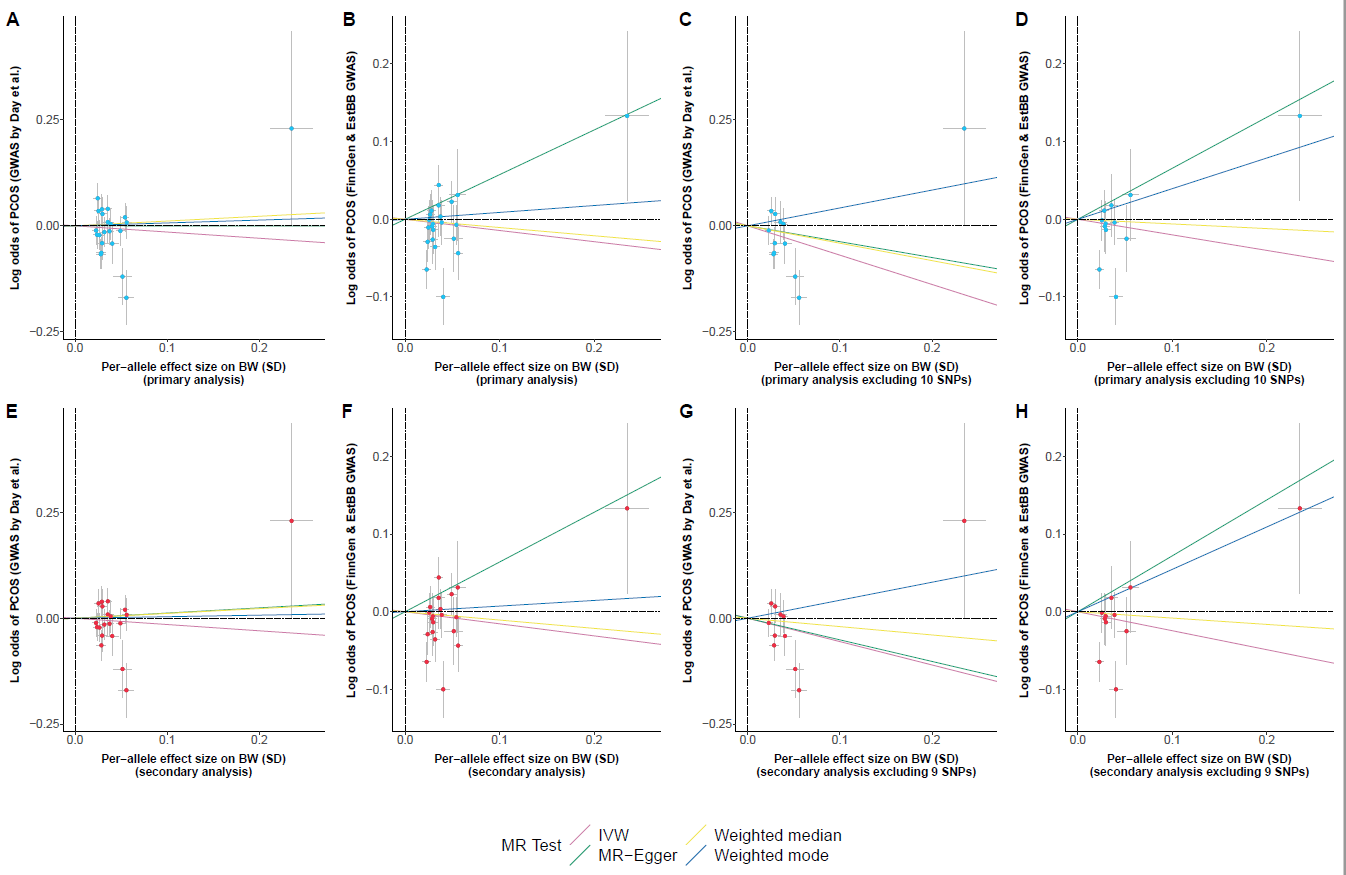


# **Supplemental** figure 2 Scatter plots visualizing the causal effect estimates of birth weight on PCOS in later life.

(A-B) scatter plots visualizing the causal effect estimates in the primary analysis using data from PCOS GWAS by Day et al. and FinnGen & EstBB PCOS GWAS for replication. (C-D) scatter plots visualizing the causal effect estimates in the primary and replication analysis after excluding 10 SNPs that were associated with potential confounders. (E-F) scatter plots visualizing the causal effect estimates in the secondary analysis using data from PCOS GWAS by Day et al. and FinnGen & EstBB PCOS GWAS for replication. (G-H) scatter plots visualizing the causal effect estimates in the secondary and replication analysis after excluding 9 SNPs that were associated with potential confounders.

SNPs that were genome-wide significantly associated with potential confounders of PCOS, including BMI, type 2 diabetes, waist/hip circumference, waist-to-hip ratio, metabolic syndrome, glucose metabolism, and lipid metabolism, were excluded from the sensitivity analyses.

BW: birth weight; EstBB: Estonian Biobank; GWAS: genome-wide association study; MR: Mendelian randomization; PCOS: polycystic ovary syndrome; SD: standard deviation; SNP: single nucleotide polymorphism.


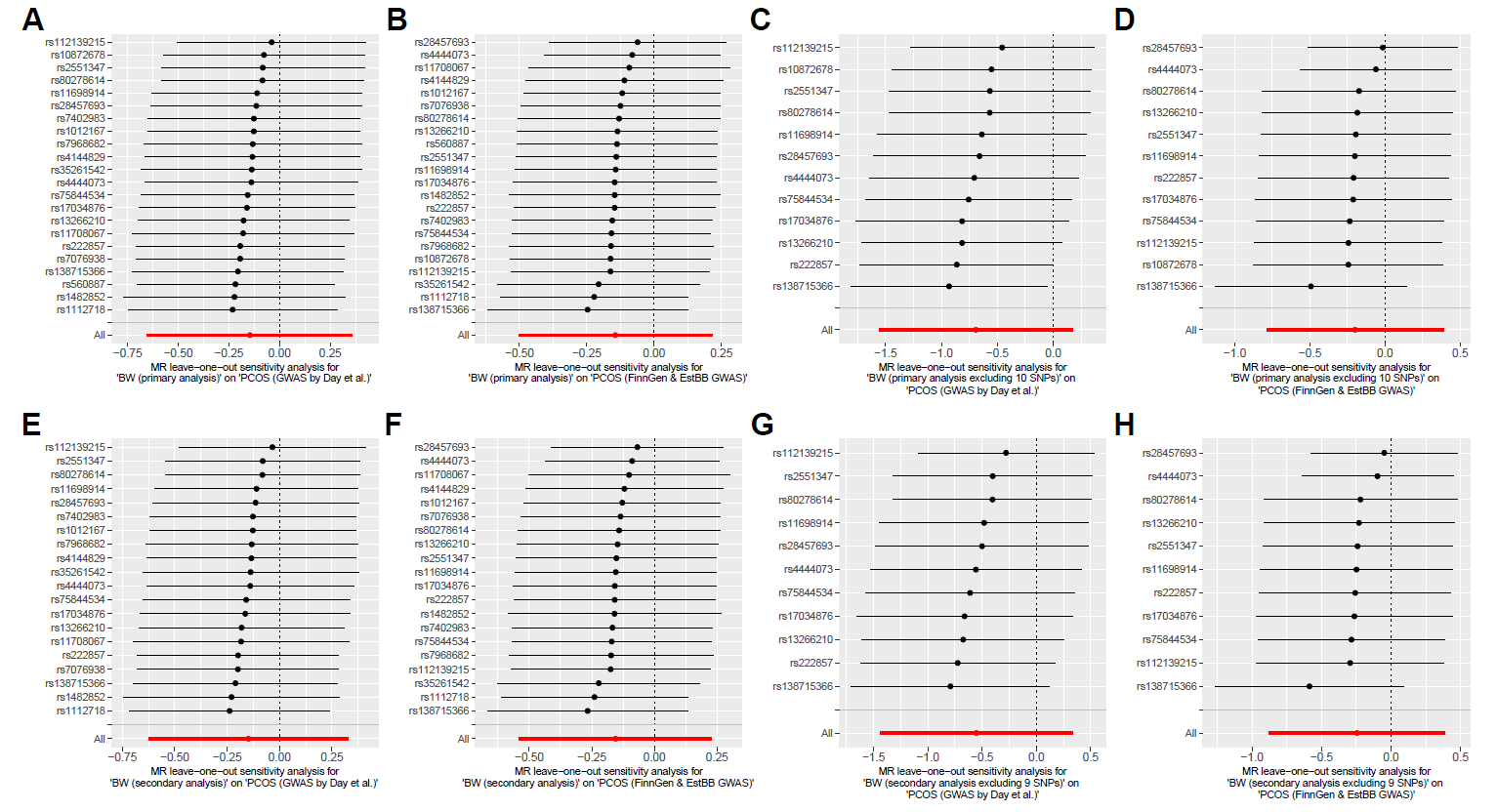


# **Supplemental** figure **3 Leave-one-out plots for the causal estimates of** **birth weight on PCOS in later life to identify strongly influential SNPs.**

(A-B) leave-one-out plots in the primary analysis using data from PCOS GWAS by Day et al. and FinnGen & EstBB PCOS GWAS for replication. (C-D) leave-one-out plots in the primary and replication analysis after excluding 10 SNPs that were associated with potential confounders. (E-F) leave-one-out plots in the secondary analysis using data from PCOS GWAS by Day et al. and FinnGen & EstBB PCOS GWAS for replication. (G-H) leave-one-out plots in the secondary and replication analysis after excluding 9 SNPs that were associated with potential confounders.

SNPs that were genome-wide significantly associated with potential confounders of PCOS, including BMI, type 2 diabetes, waist/hip circumference, waist-to-hip ratio, metabolic syndrome, glucose metabolism, and lipid metabolism, were excluded from the sensitivity analyses

BW: birth weight; EstBB: Estonian Biobank; GWAS: genome-wide association study; MR: Mendelian randomization; PCOS: polycystic ovary syndrome; SNP: single nucleotide polymorphism.
